# Differential reliance on aquatic prey subsidies influences mercury exposure in riparian arachnids and songbirds

**DOI:** 10.1002/ece3.7549

**Published:** 2021-05-01

**Authors:** Allyson K. Jackson, Collin A. Eagles‐Smith, W. Douglas Robinson

**Affiliations:** ^1^ Environmental Studies Department Purchase College SUNY Purchase NY USA; ^2^ Department of Fisheries and Wildlife Oregon State University Corvallis OR USA; ^3^ Forest and Rangeland Ecosystem Science Center U.S. Geological Survey Corvallis OR USA

**Keywords:** aquatic contaminant, aquatic‐terrestrial subsidy, mercury, riparian songbird, stable isotopes

## Abstract

Cross‐ecosystem subsidies move substantial amounts of nutrients between ecosystems. Emergent aquatic insects are a particularly important prey source for riparian songbirds but may also move aquatic contaminants, such as mercury (Hg), to riparian food webs. While many studies focus on species that eat primarily emergent aquatic insects, we instead study riparian songbirds with flexible foraging strategies, exploiting both aquatic and terrestrial prey sources. The goal in this study is to trace reliance on aquatic prey sources and correlate it to Hg concentrations in common riparian arachnids (Families Tetragnathidae, Opiliones, and Salticidae) and songbirds (Common Yellowthroat *Geothlypis trichas*, Spotted Towhee *Pipilo maculatus*, Swainson's Thrush *Catharus ustulatus*, Song Sparrow *Melospiza melodia*, and Yellow Warbler *Setophaga petechia*). We used stable isotopes of δ^13^C and δ^15^N and Bayesian mixing models in MixSIAR to determine the reliance of riparian predators on aquatic prey sources. Using mixed effects models, we found that arachnid families varied in their reliance on aquatic prey sources. While songbird species varied in their reliance on aquatic prey sources, songbirds sampled earlier in the season consistently relied more on aquatic prey sources than those sampled later in the season. For both arachnids and songbirds, we found a positive correlation between the amount of the aquatic prey source in their diet and their Hg concentrations. While the seasonal pulse of aquatic prey to terrestrial ecosystems is an important source of nutrients to riparian species, our results show that aquatic prey sources are linked with higher Hg exposure. For songbirds, reliance on aquatic prey sources early in the breeding season (and subsequent higher Hg exposure) coincides with timing of egg laying and development, both of which may be impacted by Hg exposure.

## Graphical abstract

To understand how exploiting emergent aquatic prey sources could also expose the riparian community to aquatic‐based contaminants, we used stable isotopes of δ13C and δ15N in a MixSIAR Bayesian mixing model to trace emergent aquatic prey in riparian predators (songbirds and arachnids). We determined that individual reliance on aquatic prey was positively correlated with mercury exposure, with songbirds relying more in aquatic prey early in the season. Our results show the seasonal pulse of aquatic prey to terrestrial ecosystems is likely an important source of nutrients to riparian species but may also drive season‐dependent exposure to aquatic contaminants.

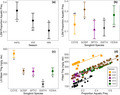

## 1 INTRODUCTION

Cross‐ecosystem nutrient subsidies are important components of ecosystem function (Polis et al., 1997). Lotic ecosystems, in particular, receive substantial allochthonous nutrient input from riparian leaf litter and detritus, and in return contribute nutrients and energy back to the surrounding riparian areas via aquatic insect emergence (Ballinger & Lake, 2006; Baxter et al., 2005). These subsidies have been shown to increase both the density and diversity of riparian predators, including lizards, birds, and bats (Fukui et al., 2006; Nakano & Murakami, 2001; Sabo & Power, 2002). In many temperate climates, terrestrial ecosystems have seasonal shifts in insect prey availability with emergent insect biomass peaking in spring, followed later by terrestrial prey after leaf‐out (Nakano & Murakami, 2001). The flux of aquatic prey is an important nutrient source, as emergent insects have higher polyunsaturated fatty acids (PUFA) than terrestrial prey (Martin‐Creuzburg et al., 2017; Moyo, 2020). Both terrestrial predatory invertebrates (e.g., arachnids) and songbirds often concentrate near aquatic habitats ostensibly to exploit the emergent insect subsidy during peak emergence times (Hagar et al., 2012; Uesugi & Murakami, 2007).

Despite the benefits of aquatic insect emergence as energetic subsidies to riparian communities, they can also degrade riparian habitats through the export of aquatically derived environmental contaminants. Many aquatic ecosystems are accumulation zones for environmental contaminants, such as mercury (Hg), other heavy metals, and polychlorinated biphenyls (PCBs). When assimilated by aquatic invertebrates, these contaminants can accompany their movement into surrounding terrestrial food webs (Jones et al., 2013; Kraus et al., 2014; Latta et al., 2015; Walters et al., 2008). As a result, riparian taxa ranging from terrestrial invertebrates to invertebrate‐eating birds and bats have been shown to accumulate aquatic‐sourced contaminants (Becker et al., 2018; Jackson, Evers, Folsom, et al., 2011; Moy et al., 2016; Yates et al., 2014).

A number of studies have assessed the influence of aquatic factors on the magnitude of emergent aquatic insect and aquatic‐derived contaminant flux into the terrestrial ecosystem (Kelly et al., 2019; Walters et al., 2008). Contaminant concentrations in aquatic insects are influenced by aquatic habitat (Jackson et al., 2019), and the biomass of insects that survive to emerge from the aquatic system can be mediated by habitat, water quality, and fish abundance (Jones et al., 2013; Paetzold et al., 2011). For some contaminants, high contaminant loading can reduce invertebrate fecundity and survival, ultimately constraining insect biomass flux and contaminant transfer to riparian food webs (Kraus, 2019; Kraus et al., 2014; Paetzold et al., 2011). Other contaminants, such as Hg, are not known to affect aquatic insect survival in Hg contaminated areas; therefore, high insect emergence rates can move large amounts of Hg into riparian zones (Tweedy et al., 2013). Mercury is of particular concern for songbirds as it continues to be found in the environment at high concentrations across North America (Cristol & Evers, 2020), and Hg exposure negatively impacts many critical aspects of the songbird life cycle, including reproduction (Brasso & Cristol, 2008; Jackson, Evers, Etterson, et al., 2011) and migration (Seewagen, 2018).

While we understand many of the aquatic factors that influence emergence of aquatic insects, less is known about how terrestrial‐based factors may play a role in riparian predator Hg exposure, especially across bird taxa. Many Hg studies focus on species, such as aerial insectivores, that feed directly and almost exclusively on emergent aquatic prey (Alberts et al., 2013; Brasso & Cristol, 2008; Custer et al., 2007). Other riparian songbirds have been shown to accumulate high levels of aquatic‐based contaminants but have much more varied exposure patterns (Jackson et al., 2015). While some variation can be explained by broad foraging guild classifications (e.g., granivore, omnivore, insectivore), there is still a large amount of unexplained variation both among species and individuals (Jackson et al., 2015; Jackson, Evers, Folsom, et al., 2011). Within species classified as insectivorous, it is assumed that individuals eat both emergent aquatic insects but also terrestrial‐based prey with little connection to the aquatic ecosystem and contaminants. Additionally, many riparian songbirds eat a variety of terrestrial invertebrate predators, such as spiders, and those invertebrate predators also can eat a varied diet consisting of both aquatic and terrestrial prey (Cristol et al., 2008; Kelly et al., 2019; Speir et al., 2014).

We hypothesize that some of the variation in Hg exposure among insectivorous predators (arachnids and songbirds) can be explained by species and individual‐level differences in prey selection, with individuals or species that rely more on aquatic prey having higher Hg exposure. Our goal in this study is to trace reliance on aquatic‐based prey and correlate it to Hg concentrations in common riparian arachnids (Families Tetragnathidae, Opiliones, and Salticidae) and songbirds (Common Yellowthroat *Geothlypis trichas*, Spotted Towhee *Pipilo maculatus*, Swainson's Thrush *Catharus ustulatus*, Song Sparrow *Melospiza melodia*., and Yellow Warbler *Setophaga petechia*). We use δ^13^C and δ^15^N stable isotopes to differentiate between aquatic and terrestrial signatures, which has been used in other studies with success (Walters et al., 2008). To answer this question using stable isotopes, we must 1) determine differences in stable isotope signatures of aquatic and terrestrial invertebrate food webs, 2) quantify differences in MeHg between aquatic and terrestrial invertebrate prey, 3) use δ^13^C and δ^15^N stable isotopes in a Bayesian mixing model determine terrestrial predator (arachnids and songbirds) reliance on the aquatic prey source, 4) evaluate seasonal changes and species‐specific differences in the proportion of aquatic prey in the diet of terrestrial predators, and 5) correlate seasonal changes in aquatic prey reliance to their Hg exposure for both arachnids and songbirds.

## 2 MATERIALS AND METHODS

### 2.1 Fieldwork

**FIGURE 1:**
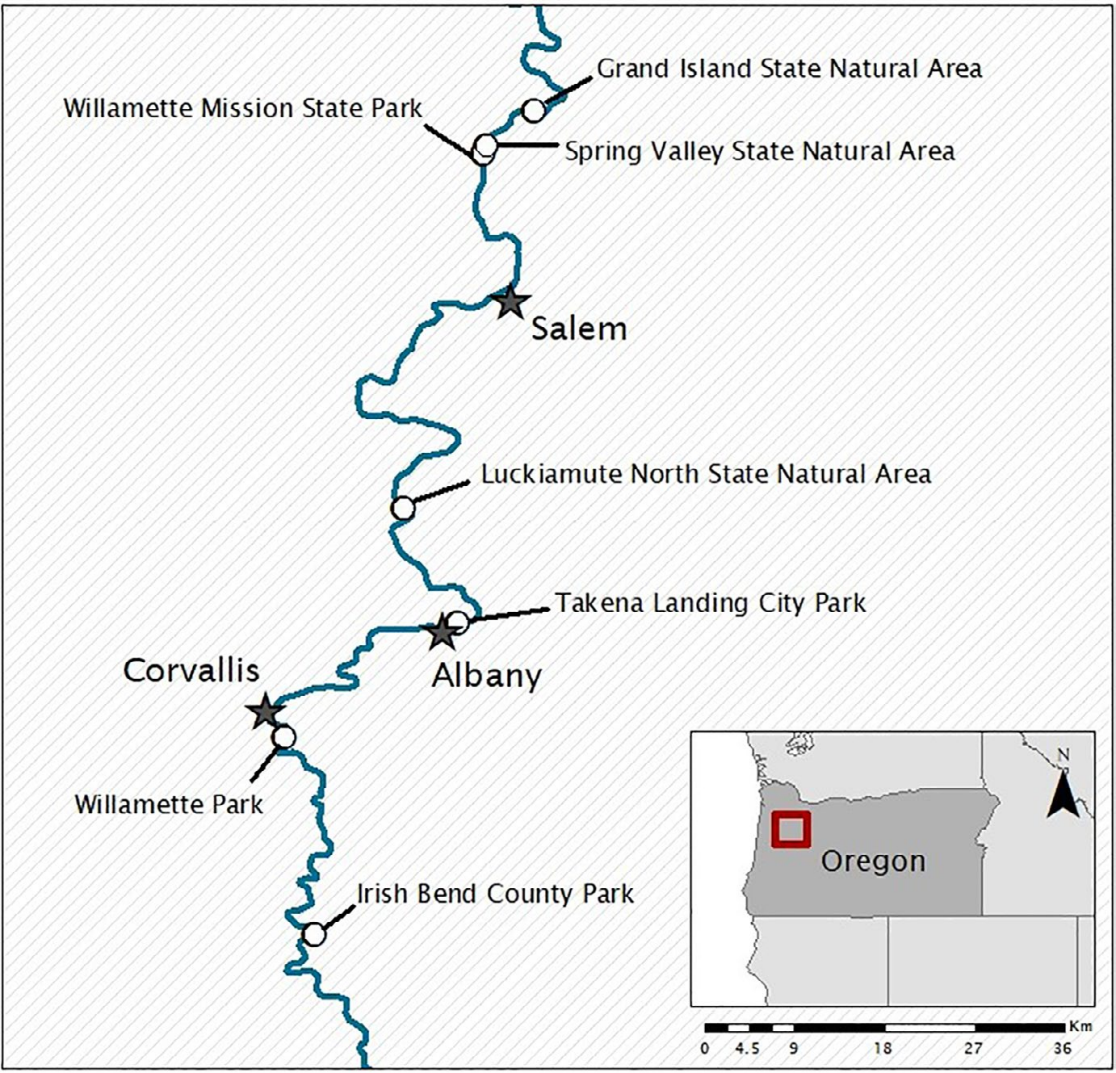
Study area along the Willamette River in western Oregon. Sites (shown as circles) had similar gallery forest terrestrial habitat paired with main channel aquatic habitat. Major cities along the Willamette are represented with stars

Field sites were chosen in riparian forest sites along the Willamette River in western Oregon (Figure 1). The Willamette River, Oregon, USA, is a major tributary to the Columbia River and drains the eastern Coast Range and western Cascades. It also has a history of anthropogenic influences, including Hg contamination (Henny et al., 2005; Hope, 2006; Hope & Rubin, 2005). The Willamette River supports a diversity of subhabitats including backwater alcoves and open channel flowing waters that differ in Hg concentrations (Jackson et al., 2019). To best differentiate aquatic and terrestrial isotope signals, we focused here on the main channel environments.

From 1 May 2013 to 23 July 2013, we sampled 7 main stem sites along the Willamette River (Figure 1). Since we are focused on birds that are not necessarily riparian obligates, we sampled songbirds living within the riparian forest using targeted mist‐netting to focus on individuals with territories within 150m of the river, the general riparian area defined in other studies (Nakano & Murakami, 2001; Walters et al., 2008). Singing individuals were identified, and mist nets (6m or 12m length, 30mm mesh) were placed opportunistically (where habitat allowed) within their territories. Playback recordings of conspecific songs were used to attract and capture riparian songbirds in a mist net. Although numerous bird species were captured and sampled, the majority (94%) of samples were of five species present at all sites: Common Yellowthroat (*Geothlypis trichas*), Spotted Towhee (*Pipilo maculatus*), Swainson's Thrush (*Catharus ustulatus*), Song Sparrow (*Melospiza melodia*), and Yellow Warbler (*Setophaga petechia*). All birds were banded with an aluminum USGS band, and any recaptures were excluded from the analyses to preserve independence of samples. Blood samples of each bird were taken from the brachial ulnar vein, using 27‐gauge needles (BD PrecisionGlide, Fisher Scientific) and heparinized microhematocrit capillary tubes (Fisherbrand, Fisher Scientific). Samples were capped with Critocaps™ (Leica Microsystems) and stored on ice in the field until they could be transferred to a freezer (within 6 hr of sampling). No more than 1% of bird's body weight of blood was collected from each individual, usually between 20 µl and 100µl.

We recorded presence of brood patch (for females) or cloacal protuberance (for males) and limited samples to individuals in breeding condition to avoid late or early migrants. We also aged each bird (Pyle, 1997) and checked for any sign of molt, limiting bird samples to only after‐hatch‐year individuals who were not molting (as an indication of postbreeding condition). All samples were collected under authority of appropriate scientific collection permits, including both State (invertebrates: Oregon DFW# 17,648; birds: Oregon DFW# 062–13) and Federal (USFWS MBTA# MB28361A; USGS Banding # 20,786) agencies. All birds were handled under approved animal care and use protocols (Oregon State University ACUP # 4,408).

We also collected aquatic and terrestrial invertebrates from sites that were collocated in space and time with the songbird sampling (Table 1). We sampled all invertebrates encountered but targeted subsequent laboratory analysis on the numerically dominant invertebrate families at each site. We did not estimate invertebrate biomass each site because our goal was focused on tracking energetic signals and not taxonomic abundance on the landscape. Aquatic invertebrates were collected via kick net and dip net in the aquatic habitat near where mist nets had been set. Terrestrial invertebrates were collected by beat sheet and sweep net in forest or shrub habitat near mist nets. All invertebrates were composited by site, transferred to glass scintillation vials, and kept on ice in the field until they could be transferred to a freezer (within 6 hr of sampling).

**TABLE 1:** Number of samples collected at each site over different sampling date ranges. δ^13^C and δ^15^N values and mercury (methylmercury in invertebrates and THg in songbirds) concentrations were determined for each sample reported here

Taxa	Group/Species	Grand Island		Irish Bend		Luckiamute			Spring Valley		Takena Landing	Willamette Mission	Willamette Park	
		June 12 – 17	July 19 – 22	May 23 – 25	July 11 – 12	April 29	June 14 – 19	July 23	May 26	July 17 – 18	May 1 – 17	June 22 ‐ July 2	June 5 – 7	Total per taxa
Invertebrates	Aquatic	5	6	3	5	3	4	5	3	4	3	4	3	48
	Emergent	4	2	4	4	3	2	2	1	4	2	4	4	36
	Emergent‐predator	1	2	1		2		3	1	1		1	2	14
	Terrestrial	4	4	3	3	2	4	4	3	3	4	6	5	45
	Terrestrial‐emergent	1	0	2	2		1		2		2		1	11
	Terrestrial‐emergent‐predator	1	1				1				1	1		5
	Terrestrial‐mixed	2	1	3	2	2	2	2	3	2	2	2	2	25
	Terrestrial predator	2	4	2	3	2	3	3	2	3	1	2	3	31
	Total invertebrate samples per date group	20	20	18	19	14	17	19	15	17	15	20	20	214
Songbirds	Common Yellowthroat	3	2	1	1		3	1				2		13
	Song Sparrow	9	2	8	2		8	2	7	3	7	4	10	62
	Spotted Towhee	2	2	1			2		1	1	2	1	2	14
	Swainson's Thrush	5	2	2	5		5	1	2	4		1	6	33
	Yellow Warbler	2	0	6	3		2						2	15
	Total bird samples per date group	21	8	18	11	0	20	4	10	8	9	8	20	137

### 2.2 Invertebrate laboratory analyses

Aquatic invertebrates were identified to family (Merritt et al., 2008), and terrestrial invertebrates were identified to either order (i.e., Hemiptera, Coleoptera, etc.) or family for arachnids (i.e., Tetragnathidae, Opiliones, Salticidae), etc. All invertebrates were composited based on the lowest taxon (order or family) identified per site and sampling date (mean, *SD*, minimum, and maximum number of individuals in the composite samples can be found in Table 2). Once composited, invertebrates were rinsed with deionized water, placed in glass vials, and dried in an oven at 50°C for a minimum of 48 hr hrs. Once dried, they were homogenized in their drying vials into a fine powder with a clean glass rod.

**TABLE 2:** Summary statistics for number of individuals included in each composite sample

Location Sampled	Invertebrate Group	Taxa (number of composited samples analyzed)	Number of individuals in the composite samples			
			Mean	*SD*	Minimum	Maximum
Aquatic	Aquatic	Amphipod (*N* = 11)	46.8	27.2	11	87
		Asian freshwater clam (*N* = 1)	12			
		Corixidae (*N* = 4)	33.5	15.8	18	52
		Crayfish (*N* = 1)	4			
		Dytiscidae (*N* = 10)	26.5	17.8	5	65
		Gyrinidae (*N* = 2)	11.0	8.5	5	17
		Hydrobiidae (*N* = 3)	30.3	25.7	15	60
		Hydrophilidae (*N* = 3)	5.0	1.7	3	6
		Lymnaeidae (*N* = 1)	30			
		Physidae (*N* = 2)	21.0	7.1	16	26
		Pleuroceridae (*N* = 10)	30.9	19.6	4	57
Aquatic	Emergent	Ephemerellidae (*N* = 6)	28.3	13.1	7	46
		Ephemeroptera composite (*N* = 1)	15			
		Glossomatidae (*N* = 3)	21.3	14.0	7	35
		Heptagenidae (*N* = 9)	56.3	24.1	22	94
		Hydropsychidae (*N* = 5)	11.4	9.7	2	25
		Leptohyphidae (*N* = 4)	20.3	13.3	6	33
		Limnephilidae (*N* = 4)	1.3	0.5	1	2
		Siphlonuridae (*N* = 3)	30.7	20.6	7	45
		Trichoptera composite (*N* = 1)	8			
Aquatic	Emergent‐predator	Aeshnidae (*N* = 2)	4.0	1.4	3	5
		Chloroperlidae (*N* = 2)	13.0	9.9	6	20
		Coenagrionidae (*N* = 3)	3.3	1.2	2	4
		Gomphidae (*N* = 3)	4.3	2.1	2	6
		Perlidae (*N* = 2)	10.0	5.7	6	14
		Perlodidae (*N* = 2)	6.5	4.9	3	10
Terrestrial	Terrestrial	Arthropod composite (*N* = 1)	18			
		Coleoptera (*N* = 11)	29.5	20.5	11	85
		Dermoptera (*N* = 1)	2			
		Hemiptera (*N* = 11)	70.4	45.9	16	170
		Hymenoptera (*N* = 2)	3.0	1.4	2	4
		Lepidoptera adult (*N* = 4)	10.0	2.9	7	14
		Lepidoptera larvae (*N* = 11)	10.8	6.8	3	20
		Orthoptera (*N* = 4)	10.5	11.6	2	27
Terrestrial	Terrestrial‐emergent	Ephemeroptera adult (*N* = 7)	12.0	11.9	3	38
		Plecoptera adult (*N* = 4)	28.8	20.6	6	52
Terrestrial	Terrestrial‐emergent‐predator	Coenagrionidae (*N* = 5)	3.8	3.1	1	8
Terrestrial	Terrestrial‐mixed	Arthropod composite (*N* = 4)	9.8	4.0	4	13
		Diptera (*N* = 12)	27.5	20.8	7	68
		Neuroptera (*N* = 7)	12.4	22.0	1	62
		Tipulidae (*N* = 2)	2.5	2.1	1	4
Terrestrial	Terrestrial predator	Opiliones (*N* = 6)	6.7	4.9	2	14
		Salticidae (*N* = 3)	7.0	2.6	5	10
		Spider composite (*N* = 11)	61.9	44.0	14	174
		Tetragnathidae (*N* = 10)	18.0	13.4	4	43

Methylmercury (MeHg) is the bioavailable form of Hg that biomagnifies through trophic levels; inorganic Hg does not biomagnify. Of the Hg in blood of most birds, 95%–99% is MeHg (Rimmer et al., 2005). Percentages of MeHg in invertebrates have been shown to vary considerably (Riva‐Murray et al., 2020). We therefore analyzed MeHg concentrations in all invertebrate samples following EPA method 1,630 (U.S. Environmental Protection Agency, 2001) at the USGS FRESC contaminant ecology research laboratory in Corvallis, OR. Briefly, 2–10 mg of dried tissue homogenate was digested in 3‐4ml 30% nitric acid at 60^◦^C overnight (~15 hr), ethylated with 1% sodium tetraethylborate, then analyzed via cold vapor atomic fluorescence spectrometry on a MERX‐*M* (Brooks Rand Instruments, Seattle, Washington, USA) automated methylmercury analyzer. Quality assurance measures included analysis of two independently derived liquid calibration standards, two certified reference materials (scallop tissue [IAEA‐452; International Atomic Energy Agency, Vienna, Austria] or lobster hepatopancreas [TORT‐3; National Research Council of Canada, Ottawa, Canada]). Percent recoveries averaged 99% (*SD* =8%) for 10pg MeHg standard, 96% (*SD* =13%) for 100pg MeHg standard, 99% (*SD* =21%) for IAEA‐452 averaged and 86% (*SD* =5%) for TORT‐3.

### 2.3 Songbird blood analysis

Songbird whole blood samples were not composited, but instead run on an individual basis as dry weight. Because 95%–99% of Hg in bird blood is MeHg (Rimmer et al., 2005), we analyzed bird blood for total Hg using a Milestone tri‐cell DMA‐80 Direct Hg Analyzer (Milestone, Shelton, Connecticut USA) at the USGS CERL. This method uses combustion and gold amalgamation coupled with cold vapor atomic absorption spectrometry following US Environmental Protection Agency method 7,473. Certified reference material (dogfish muscle tissue [DORM‐4; National Research Council of Canada, Ottawa Canada] and lobster hepatopancreas tissue [TORT‐2; TORT‐3; National Research Council of Canada, Ottawa Canada]), calibration verification (liquid standards), CRM duplicates, air blanks, and boat blanks were included with each run. Total mercury analysis QA/QC included recoveries of 99.8% (*SD*=8.4%, *N* = 67) for calibration verification, recovery of 98% (SE =11.5%, *N* = 100) for certified reference material, and absolute percent difference of 2% (*SD* =3%, *N* = 43) for duplicates.

### 2.4 Stable isotope analysis

Composited, dried, and homogenized invertebrates and dried whole blood from individual birds were packed in tin capsules for stable isotope analysis at the University of California, Davis Stable Isotope Facility. All samples were analyzed using a PDZ Europa ANCA‐GSL elemental analyzer interfaced to a PDX Europa 20–20 isotope ratio mass spectrometer (Sercon Ltd., Cheshire, UK). Stable isotope values are reported as delta (δ) values using the equation ‰ = [(R_sample_ / R_standard_) −1] *1,000 where R = the ratio of the heavy isotope to the light isotope. Nitrogen samples were standardized against N_2_ in air, and carbon isotopes were standardized against Vienna PeeDee Belemnite. Based on replicate analysis of standard reference materials, we calculated the instrument standard for invertebrates and bird blood separately. For invertebrates, reference materials included bovine liver (δ^13^C *SD* =0.08, δ^15^N *SD* =0.29), USGS‐41 glutamic acid (δ^13^C *SD* =0.14, δ^15^N *SD* =0.15), nylon‐5 (δ^13^C *SD* =0.05, δ^15^N *SD* =0.17, and glutamic acid (δ^13^C *SD* =0.1, δ^15^N *SD* =0.2). For bird blood, reference materials included bovine liver (δ^13^C *SD* =0.85, δ^15^N *SD* =0.24), USGS‐41 glutamic acid (δ^13^C *SD* =0.29, δ^15^N *SD* =0.31), nylon‐5 (δ^13^C *SD* =0.34, δ^15^N *SD* =0.26, and glutamic acid (δ^13^C *SD* =0.3, δ^15^N *SD* =0.64).

### 2.5 Statistical analysis

All statistical modeling was conducted with Program R (Version 4.0.3 “Bunny‐Wunnies Freak Out,” R Foundation for Statistical Computing). We grouped invertebrates into broad categories based on diet and life history. Aquatic‐collected samples were grouped into aquatic (fully aquatic life stage, no emergent life stage; e.g., snails), emergent (larval emergent aquatic insects with herbivorous or omnivorous feeding habits; e.g., Trichoptera), and emergent predators (larval emergent aquatic insects with entirely predatory feeding habits; e.g., Odonata). Terrestrially collected samples were grouped into terrestrial (primarily herbivorous feeding habit with no aquatic life stage; e.g., Hemiptera), terrestrial‐emergent (adult stage of emergent aquatic insects, those that do not feed as adults; e.g., Ephemeroptera), terrestrial‐emergent predators (adult life stage of emergent aquatic insects, those that are predators as adults; e.g., Zygoptera), and terrestrial‐mixed (terrestrial insect orders that have both aquatic and terrestrial larval stages; e.g., Diptera). The terrestrial‐mixed category is necessary because we only identified terrestrial insects to order, and some of the orders have mixed life history strategies (Table 3). Arachnids and songbirds were not included in these groups, as they were the consumers of interest for the stable isotope models to follow.

**TABLE 3:** Summary statistics stable isotopes and MeHg for invertebrate taxa and groups. Different letters moving down a column indicate statistically significant differences between groups in δ^13^C or δ^15^N

Location Sampled	Invertebrate Group	Taxa	*N*	Mean δ^13^C	*SD* δ^13^C	Mean δ^15^N	*SD* δ^15^N	Geomean Hg	Hg Back‐transformed SE
Aquatic	Aquatic invertebrate, no emergent life stage	**Aquatic overall**	**48**	**−22.122^c^**	**2.335**	**10.623^c^**	**2.295**	**72.013**	**19.900**
		Amphipoda	11	−21.125	1.470	11.641	2.892	54.188	10.299
		Asian freshwater clam	1	−25.530		8.290		127.000	
		Corixidae	4	−23.683	3.637	11.198	1.680	120.548	17.216
		Crayfish	1	−20.770		10.570		69.300	
		Dytiscidae	10	−21.716	2.631	10.548	2.286	100.242	28.364
		Gyrinidae	2	−22.975	1.959	8.795	0.247	74.871	33.164
		Hydrobiidae	3	−23.277	1.410	8.520	0.269	66.464	14.899
		Hydrophilidae	3	−23.730	1.011	10.353	0.187	59.807	3.846
		Lymnaeidae	1	−21.160		7.530		22.700	
		Physidae	2	−25.090	5.303	9.220	1.245	61.070	22.411
		Pleuroceridae	10	−21.296	1.506	11.255	2.373	68.060	23.572
Aquatic	Larval emergent aquatic insects (those with herbivorous or omnivorous feeding habits)	**Emergent overall**	**36**	**−21.937^c^**	**2.707**	**10.519^c^**	**2.740**	**26.816**	**9.143**
		Ephemerellidae	6	−21.468	3.146	10.650	2.895	22.760	5.395
		Ephemeroptera composite	1	−19.810		13.040		24.600	
		Glossomatidae	3	−21.563	2.746	9.613	1.718	8.742	1.246
		Heptagenidae	9	−21.667	1.824	11.183	2.524	23.974	5.367
		Hydropsychidae	5	−22.130	2.343	9.570	2.132	49.497	12.720
		Leptohyphidae	4	−20.555	1.337	8.923	2.615	63.091	16.767
		Limnephilidae	4	−22.543	3.326	9.548	4.156	13.293	2.693
		Siphlonuridae	3	−26.117	4.036	11.430	1.896	59.896	21.592
		Trichoptera composite	1	−20.030		16.220		14.000	
Aquatic	Larval emergent aquatic insects (those with entirely predatory feeding habits)	**Emergent predators overall**	**14**	**−21.960^c^**	**1.606**	**12.428^c^**	**3.975**	**71.720**	**27.075**
		Aeshnidae	2	−22.965	2.284	10.505	0.700	203.499	0.307
		Chloroperlidae	2	−20.420	1.739	13.900	0.976	26.450	2.993
		Coenagrionidae	3	−22.910	2.332	10.450	0.921	124.141	33.318
		Gomphidae	3	−21.327	0.505	15.000	8.660	112.572	2.445
		Perlidae	2	−21.855	1.761	13.515	0.884	42.220	13.588
		Perlodidae	2	−22.125	0.262	10.900	1.047	26.000	4.190
Terrestrial	Terrestrial insects (primarily herbivorous with no aquatic larval stage)	**Terrestrial overall**	**45**	**−28.019^a^**	**1.553**	**3.159^a^**	**2.124**	**2.151**	**1.129**
		Arthropod composite	1	−27.240		2.650		23.500	
		Coleoptera	11	−27.263	1.168	3.358	1.524	3.766	1.749
		Dermoptera	1	−27.510		2.130		1.440	
		Hemiptera	11	−27.641	1.017	2.961	1.346	1.579	0.868
		Hymenoptera	2	−25.960	0.071	6.045	3.429	2.862	3.162
		Lepidoptera adult	4	−27.625	2.210	5.600	3.742	7.997	2.932
		Lepidoptera larvae	11	−29.568	1.407	2.042	1.684	0.935	0.162
		Orthoptera	4	−28.620	0.813	2.730	2.446	1.512	0.388
Terrestrial	Terrestrial life stage of emergent aquatic insects (those that do not feed as adults)	**Terrestrial‐emergent overall**	**11**	**−22.328^c^**	**0.761**	**12.029^c^**	**2.055**	**25.656**	**4.865**
		Ephemeroptera adult	7	−22.479	0.661	11.504	2.073	22.945	5.129
		Plecoptera adult	4	−22.065	0.954	12.948	1.926	31.193	2.174
Terrestrial	Terrestrial life stage of emergent aquatic insects (those that are predators as adults)	**Coenagrionidae**	**5**	**−28.520^ab^**	**3.436**	**9.806^c^**	**1.816**	**59.118**	**23.709**
Terrestrial	Terrestrial orders that have both aquatic and terrestrial larval stages	**Terrestrial‐mixed overall**	**25**	**−25.963^b^**	**1.553**	**6.150^b^**	**2.575**	**14.722**	**5.175**
		Arthropod comp.	4	−26.708	1.697	4.933	3.880	20.630	7.097
		Diptera	12	−26.349	0.641	6.605	1.655	16.353	6.228
		Neuroptera	7	−24.781	2.186	6.470	2.894	10.120	3.424
		Tipulidae	2	−26.295	1.435	4.740	4.525	14.825	2.635

#### 2.5.1 Prey sources

To determine suitable stable isotope endmembers for our analysis, we first explored differences in δ^13^C and δ^15^N between our invertebrate groups. We used a one‐way analysis of variance (ANOVA) followed by Tukey's HSD test to quantify differences in both δ^13^C and δ^15^N among the invertebrate groups. We also compared MeHg concentrations among the invertebrate groups (one‐way ANOVA on log‐transformed MeHg concentrations).

Based on these analyses, we designated the “emergent” insect group as being representative of the aquatic prey source and the “terrestrial” insect group as representative of the terrestrial prey source (groups explained above and in Table 3). When we refer to either aquatic or terrestrial prey sources, we are strictly indicating only the stable isotope signal came from those different endmembers, but we do not necessarily know the exact prey the consumers ate. Thus, the stable isotopes results would only be distinguishing if the arachnids or songbirds were receiving energy that originated in those respective habitats and would not inform that they were directly consuming emergent aquatic insects.

#### 2.5.2 MixSIAR Bayesian models

We used package MixSIAR (Bayesian Mixing Models in R, version 3.1.12) to estimate the proportion of the aquatic prey source in the diet of both riparian arachnids and songbirds. Prey sources supplied to MixSIAR were the same for both arachnid and songbird models. Aquatic prey (*N* = 36) had mean δ^13^C = −21.937 (*SD* =2.707) and mean δ^15^N = 10.519 (*SD* =2.740). Terrestrial prey (*N* = 45) had mean δ^13^C = −28.019 (*SD* =1.553) and mean δ^15^N = 3.159 (*SD* =2.124). We chose trophic discrimination factors (TDF) based on available data for species with similar feeding habits and prey sources. We used different TDF for arachnids (from Graf et al., 2020; *Tetragnatha sp*. and *Pardosa sp*., separate TDF for aquatic (δ^13^C = 0.5 + 0.19 *SD*, δ^15^N = 2.3 + 0.24) and terrestrial sources (δ^13^C = 0.4 + 0.17 *SD*, δ^15^N = 2.3 + 0.28 *SD*) and songbirds (from Herrera and Reyna 2007; *Habia fuscicauda*, red‐throated ant‐tanager, whole blood, δ^13^C = 2.2 + 0.1, δ^15^N = 2.6 + 0.2). No TDF exists for the species sampled in this study, but we also ran our MixSIAR results using TDF for songbirds used in other studies (Michelson et al., 2018) and found no difference in results.

The arachnid model included individual sample composite as a random effect, uninformative priors, a residual*process error structure and used the “long” run length to achieve model convergence (chain length = 300,000, burn‐in = 200,000, thin = 100, # chains = 3). The songbird model included individual bird as a random effect, uninformative priors, a residual*process error structure and used the “very long” run length to achieve model convergence (chain length =1,000,000, burn‐in = 500,000, thin = 500, # chains = 3). We assessed model convergence using Gelman‐Rubin and Geweke diagnostics.

#### 2.5.3 Mixed effects models

We used the mixSIAR‐calculated proportion aquatic prey source for both arachnids and songbirds in all subsequent analysis. We developed mixed effects models using packages lme4 (Bates et al., 2019) and lmerTest (Kuznetsova et al., 2019) to test factors influencing aquatic prey reliance and Hg exposure. For each test, we first developed a global model that included all main effects and associated two‐way interactions. If an interaction had a p‐value >0.1, we excluded it and reran the model without it. To illustrate changes in proportion aquatic prey and Hg concentrations through the season for any models with significant interactions that included Julian date (date of sampling, coded as number of days since 1 January), we compared model‐estimated least squares means (package emmeans; Lenth, 2019) at three time points in the season. These points were representative of early season (Julian date = 120 = April 30), mid‐season (Julian date =160 = June 9), and late season (Julian date = 200 = July 19).

First, for arachnids, we ran a mixed effects model that accounted for site as a random effect to determine if date, group (Tetragnathidae, Salticidae, Opiliones, or spider composite), or an interaction of date by group influenced proportion of aquatic prey of these arachnids. The interaction was not significant so we report the reduced model with only main effects. We then ran models to determine if proportion of aquatic prey and arachnid group influenced MeHg exposure, including site as a random effect.

Second, for riparian songbirds, we ran a mixed effects model that accounted for site as a random effect to determine if date, songbird species (Common Yellowthroat, Spotted Towhee, Swainson's Thrush, Song Sparrow, and Yellow Warbler), or an interaction of date and species influenced proportion of aquatic prey in the blood of riparian songbirds. The interaction was not significant, and so we report the reduced model with only main effects. We then ran a mixed effects model to determine if proportion aquatic prey, species, or an interaction of proportion aquatic prey and species influenced blood THg concentration in songbirds.

## 3 RESULTS

### 3.1 Analysis of prey sources

**FIGURE 2:**
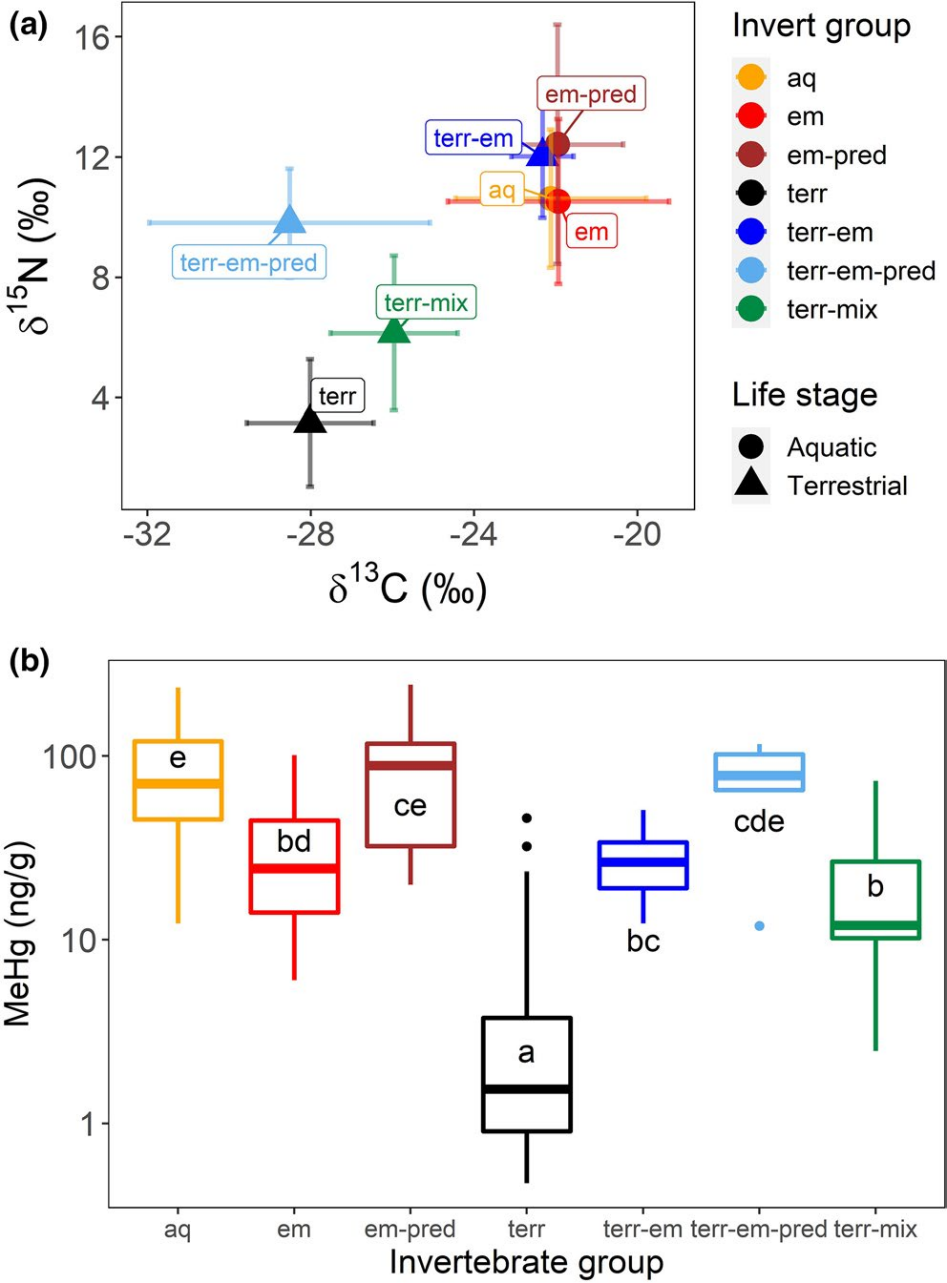
(a) Stable isotopes of carbon‐13 and nitrogen‐15 in invertebrate groups, based on sampling location (aquatic=circles or terrestrial =triangles). Biplots indicate mean and *SD*. Samples that make up each mean and *SD* can be found in Table 1. (b) MeHg differences between invertebrate groups. Different letters indicate statistically significant differences (*p* <.05). Aquatic‐collected samples were grouped into aquatic (aq; fully aquatic life stage, no emergent life stage, *N* = 48), emergent (em; larval emergent aquatic insects with herbivorous or omnivorous feeding habits, *N* = 36), and emergent predators (em‐pred; larval emergent aquatic insects with entirely predatory feeding habits, *N* = 14). Terrestrially collected samples were grouped into terrestrial (terr; primarily herbivorous feeding habit with no aquatic life stage, *N* = 45), terrestrial‐emergent (terr‐em; adult stage of emergent aquatic insects, those that do not feed as adults, *N* = 11), terrestrial‐emergent predators (terr‐em‐pred; adult life stage of emergent aquatic insects, those that are predators as adults, *N* = 5—all Coenagrionidae), and terrestrial‐mixed (terr‐mix; terrestrial insect orders that have both aquatic and terrestrial larval stages, *N* = 25)

We analyzed a total of 214 invertebrate samples for δ^13^C, δ^15^N, and MeHg and 137 songbird individual samples for δ^13^C, δ^15^N, and THg (Table 1). We first compared stable isotope signatures of the invertebrate groups in both aquatic and terrestrial food webs (Figure 2a). Invertebrates sampled from the terrestrial environment were generally depleted in both δ^13^C and δ^15^N compared to invertebrates sampled from the aquatic environment. One notable exception was emergent insects caught as terrestrial nonfeeding adults (mayflies and stoneflies); their isotope signatures were similar to the aquatic environment. Overall and taxa‐specific means and *SD* for δ^13^C, δ^15^N, and geometric means and back‐transformed SE for MeHg can be found in Table 3. We found a significant difference in both δ^13^C (one‐way ANOVA, *F* = 51.3, *p* <.001) and δ^15^N (one‐way ANOVA, *F* = 56.0, *p* < .001) between invertebrate groups. Tukey pairwise analysis revealed consistent differences (*p* <.01) between terrestrial insects and aquatic, emergent aquatic, and emergent aquatic predators in both δ^13^C and δ^15^N (results from all pairwise comparisons are included in Table 3).

Invertebrate groups also differed in MeHg concentrations (one‐way ANOVA on log‐transformed MeHg, *F* = 73.2, *p* <.001, Figure 2b). Pairwise comparisons (Tukey HSD, *p* <.001) indicated that terrestrial insects were significantly lower in MeHg than all other groups (including both aquatic invertebrates and emergent insects caught in both their aquatic and terrestrial life stages). Although there was no difference in δ^13^C or δ^15^N between aquatic invertebrates and emergent aquatic invertebrates, there was a significant difference in MeHg between these groups.

### 3.2 MixSIAR Bayesian isotope mixing models

**FIGURE 3:**
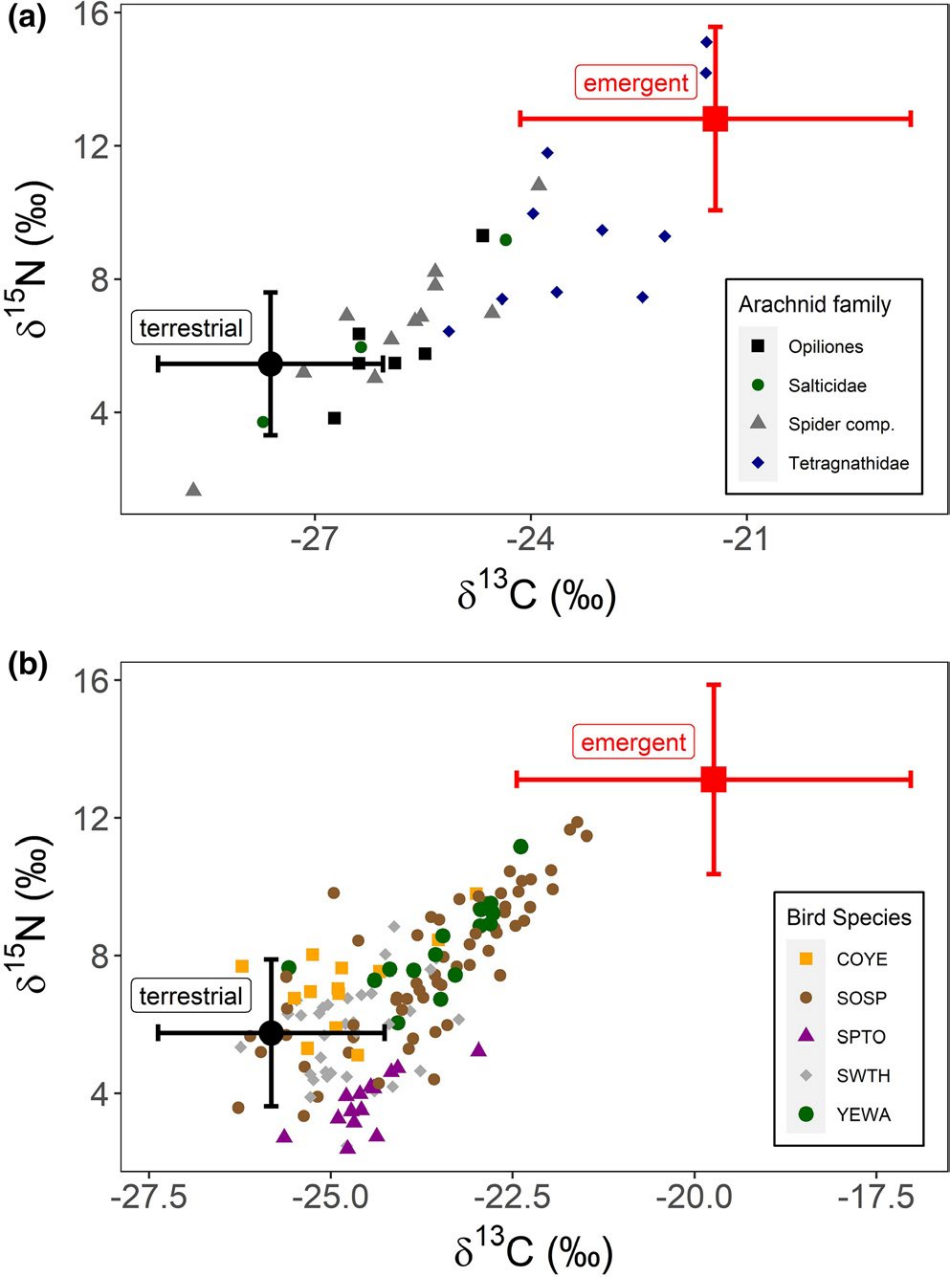
Isospace plots for stable isotopes of δ13C and δ15N in A) arachnids and B) riparian songbirds. Prey sources (shown as mean plus error bars) are corrected for trophic enrichment factors based on Graf et al. 2020 for invertebrates and Herrera and Reyna 2007 for songbirds (values in methods) using the following equations: source mean =meansource + meandis; sourcesd = √(SDsource2 + SDdisc2)

We used emergent aquatic insects as our aquatic prey source (δ^13^C = −21.937 + 2.707SD, δ^15^N = 10.519 + 2.740SD, *N* = 36) and terrestrial insects as our terrestrial prey source (δ^13^C = −28.019 + 1.553SD, δ^15^N = 3.159 + 2.124SD, *N* = 45) for MixSIAR analysis for riparian arachnids and birds (Figure 3). When source data were corrected for discrimination factors, isotopes values for both arachnid (*N* = 30, Figure 3a) and avian (*N* = 137, Figure 3b) predators fell between the aquatic and terrestrial prey sources.

### 3.3 Arachnids

**FIGURE 4:**
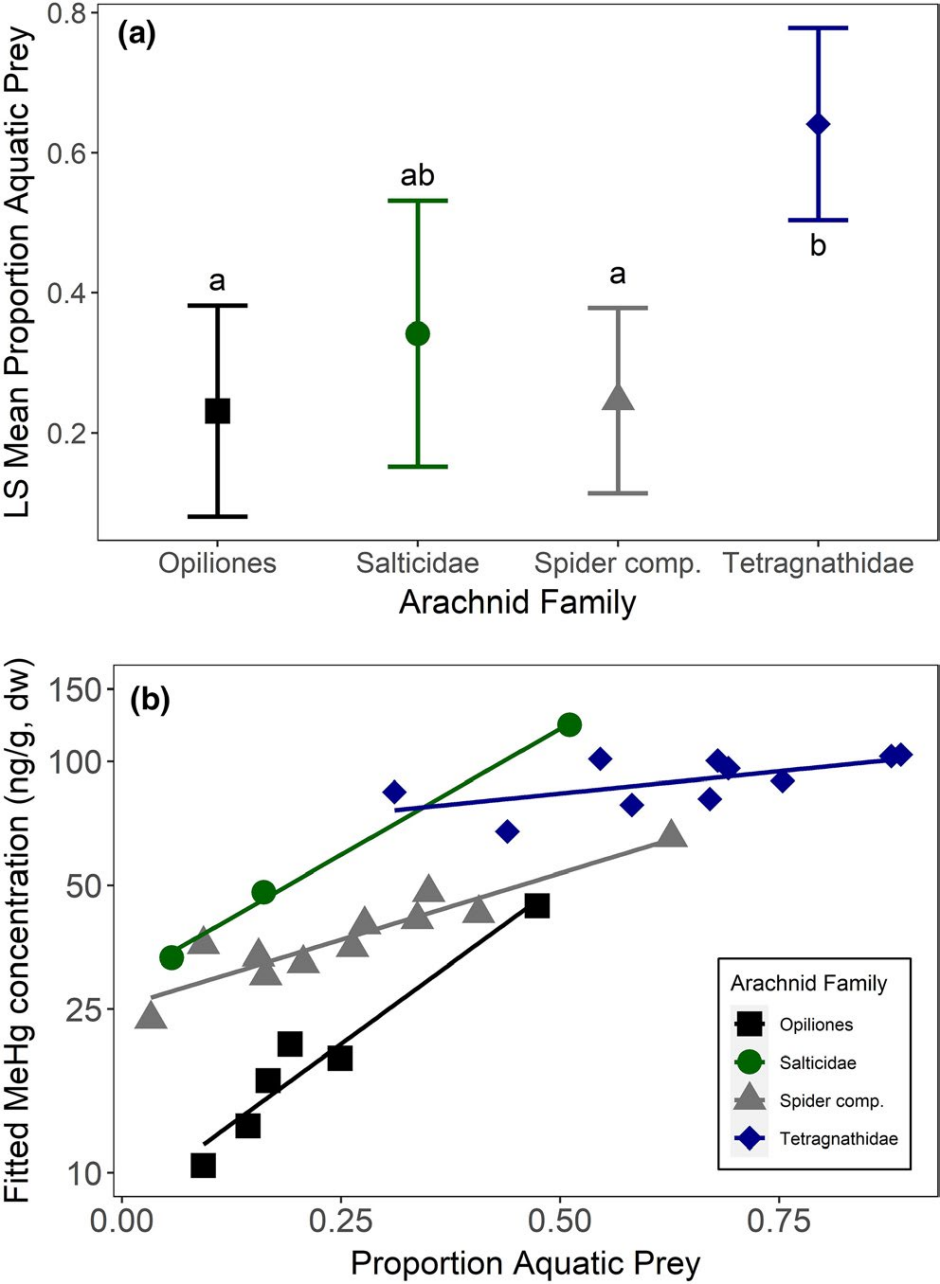
(a) Significant interaction of arachnid group in a model to predict proportion of aquatic prey in the diet of arachnids, after accounting for site differences. Different letters indicate statistically significant differences (*p* <.05). (b) Relationship between proportion aquatic prey and MeHg concentration in arachnids, after accounting for site differences

Using the results for proportion aquatic prey as the dependent variable in a mixed effects model with site as a random effect, we found that proportion aquatic prey differed among arachnid groups (*F* = 22.5, *p* <.001, Figure 4a) but not over time (*F* = 1.15, *p* =.29). We next used MeHg concentration as the dependent variable and found that MeHg concentrations also differed among arachnid groups (Opiliones, Salticidae, Tetragnathidae, arachnid composite; *F* = 6.85, *p* =.002) and was positively related to proportion aquatic prey (*F* = 25.71, *p* <.0001). However, the interaction between proportion of aquatic prey and arachnid family (*F* = 3.51, *p* =.035) indicated that the slopes for the relationship between proportion of aquatic prey and MeHg concentrations varied among taxa (Figure 4b).

### 3.4 Terrestrial songbird predators

**FIGURE 5:**
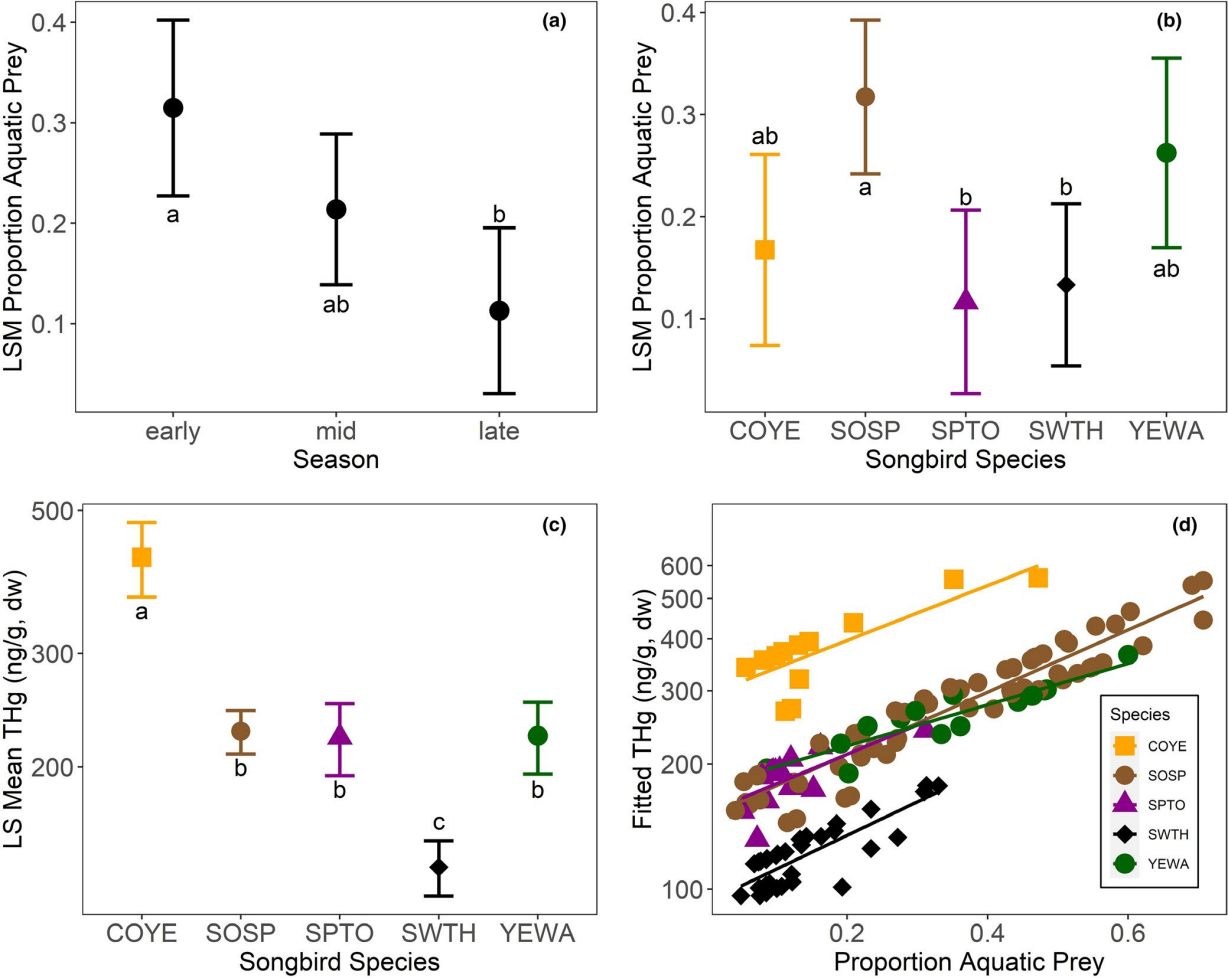
Riparian songbird model results. Different letters indicate statistically significant differences (*p* <.05). (a) Date influences proportion aquatic prey in the diet of songbirds; aquatic prey declines later in the season. Early group =Julian date 120 (April 30); mid group =Julian date 160 = June 9); late group =Julian date 200 (July 19). (b) Species influences proportion aquatic prey in diet: Common Yellowthroat (COYE), Song Sparrow (SOSP), Spotted Towhee (SPTO), Swainson's Thrush (SWTH), and Yellow Warbler (YEWA). C) Species differences in THg concentrations. D) Relationship between proportion aquatic prey and THg concentration in songbird species

We ran similar models for riparian songbirds (Common Yellowthroat, Song Sparrow, Spotted Towhee, Swainson's Thrush, and Yellow Warbler). Proportion aquatic prey in bird diets was influenced by date (*F* = 16.96, *p* <.0001) and was different among species (*F* = 15.67, *p* <.0001). Throughout the season, proportion of aquatic prey in the songbird diet declined, from 31.5% (SE = 4.2%) at the early sampling period (Julian day 120 = Apr 30) to 11.3% (SE = 3.8%) at the late sampling point (Julian day 200 = July 19) (Figure 5a). Song Sparrows (least squares mean =31.7%, SE =3.2%) showed higher reliance on aquatic prey than Swainson's Thrush (least squares mean = 13.3%, SE = 3.6%) or Spotted Towhee (least squares mean = 11.7%, SE = 3.6%) (Figure 5b).

THg concentrations in riparian songbirds were positively correlated with proportion aquatic prey (*F* = 27.9, *p* <.0001) and differed among species (*F* = 12.3, *p* <.0001), varying 3‐fold between the species with the lowest and highest concentrations. Swainson's Thrush (140 ng/g, SE =7.03) had the lowest least squares mean THg concentration, followed by Spotted Towhee (222 ng/g, SE =14.5), Yellow Warbler (224 ng/g, SE =14.5), Song Sparrow (227 ng/g, SE =9.01), and Common Yellowthroat (423 ng/g, SE =28.63) (Figure 5c). Across species, blood THg concentrations were positively correlated with proportion aquatic prey (Figure 5d).

## 4 DISCUSSION

Aquatic productivity provides important energetic subsidies to surrounding terrestrial communities (Baxter et al., 2005; Nakano & Murakami, 2001), and our findings are consistent with this body of literature. We show that for main channel sites in the Willamette River, δ^13^C and δ^15^N isotopes can be used to trace the amount of aquatic prey in the diet of riparian predators. We found that aquatic energy subsidies comprised a large component of riparian predator diets, and species in riparian habitats foraging on aquatic‐derived food items were more likely to have higher Hg exposure than those foraging on prey derived from terrestrial habitats. Contaminants like Hg are generally higher in aquatic than terrestrial environments; thus, these subsidies may represent substantial vectors of aquatic contaminants into terrestrial communities (Kraus et al., 2014; Moyo, 2020; Walters et al., 2008).

Seasonal pulses of emergent aquatic invertebrates into terrestrial ecosystems have been identified in many different habitats (Ballinger & Lake, 2006; Bartrons et al., 2015; Baxter et al., 2005). Seasonal weather shifts in the Pacific Northwest, from wet spring through dry summer months, which span the songbird breeding season, can influence terrestrial invertebrate abundance as well as emergence pulses of aquatic invertebrates (Nakano & Murakami, 2001). Using isotopic signatures, we found that songbirds shifted from being largely reliant on aquatic‐sourced prey to a greater reliance on terrestrially sourced prey. Songbird reliance on aquatic‐sourced prey declined throughout the season, presumably due to the later emergence of terrestrial invertebrates following the leafing out of deciduous vegetation. Our findings are supported by previous studies that have used field observations to determine reliance on terrestrial versus aquatic prey in riparian songbirds (Nakano & Murakami, 2001; Uesugi & Murakami, 2007).

For arachnids, MeHg concentrations were also correlated with aquatic prey reliance but the relationship between reliance on aquatic prey and MeHg exposure varied among arachnid families. For example, tetragnathid spiders, often used in contaminant studies (Beaubien et al., 2019; Otter et al., 2013; Speir et al., 2014; Sullivan et al., 2016; Walters et al., 2008, 2010), had relatively high reliance on aquatic prey and similar MeHg concentrations, indicating their diet is fairly constrained along the Willamette River. Previous studies support this finding that riparian tetragnathids rely heavily on aquatic resources (Ortega‐Rodriguez et al., 2019; Speir et al., 2014). Other arachnid families are much more mobile with various hunting strategies (Ortega‐Rodriguez et al., 2019), and we found that they varied in both their reliance on aquatic prey and their MeHg exposure. This has important implications for predicting biomagnification of aquatic contaminants through the terrestrial food web (Kraus, 2019); risk cannot be assessed based on arachnid family MeHg concentrations alone, but instead must take into account density and relative numbers of each family available to riparian songbirds. While we focused on one habitat type in one river system, variation in the surrounding habitat especially urbanization gradients may increase the proportion of aquatic prey in the diet of spiders (Kelly et al., 2019).

Riparian songbirds exhibited similar taxonomic variation in their reliance on aquatic prey, with Song Sparrows and Yellow Warblers more reliant on aquatic prey than Spotted Towhees, Swainson's Thrushes, and Common Yellowthroats. Perhaps more importantly, our study demonstrated that individuals of each species ranged from low to high use of aquatic prey. Use of aquatic subsidies at an individual level can benefit a variety of health metrics including migratory refueling (MacDade et al., 2011) and nestling growth rates (Dodson et al., 2016). Contrary to these benefits, our study also shows that reliance on aquatic prey increases Hg exposure, which can interfere with migration (Seewagen, 2018), reproduction (Jackson, Evers, Etterson, et al., 2011; Varian‐Ramos et al., 2014), and survival (Ma et al., 2018) at environmentally relevant exposures. The varying reliance on aquatic prey among species and individuals complicates the calculation of Hg risk to riparian communities near contaminated water bodies because a more detailed understanding of foraging ecology (beyond broad classification of granivore, omnivore, or insectivore) is needed to assess risk. Despite that all of the songbirds in this study are reported to be insectivorous during the breeding season, they still differed in the relative contributions of aquatic and terrestrial sourced prey, which directly influenced their Hg exposure.

Birds and arachnids that rely more on aquatic prey have higher Hg exposure, which follows findings of others who have shown that emergent aquatic insects are an important source of aquatic contaminants to terrestrial ecosystems (Chumchal & Drenner, 2015; Speir et al., 2014; Walters et al., 2008, 2010). Very few studies have quantified individual or species‐specific reliance on aquatic prey outside of aerial insectivores, which are known to focus almost entirely on emergent insects (Alberts et al., 2013; Brasso & Cristol, 2008; Custer et al., 2008). Our expanded effort on other forest riparian songbirds indicated that species with more flexible foraging strategies demonstrated more plastic reliance on prey source over time. This study provides an example of how individual‐level factors in species foraging ecology influence variation in mercury exposure. Moreover, we did not find evidence that proximity of nesting territory (inferred from capture location) to water was the sole determinant of aquatic energy to riparian songbird diet.

The Hg levels we measured in songbirds were below general thresholds thought to cause reproductive harm (Jackson, Evers, Etterson, et al., 2011; Varian‐Ramos et al., 2014, p.). It is important to understand, however, that these thresholds are developed for a limited number of species, none of which were sampled in this project. It is likely that species and individuals vary in their sensitivity to Hg, and so taxa‐wide threshold levels should be used with caution (Varian‐Ramos et al., 2014, p.). While the Hg levels are relatively low, our findings related to habitat, species, and season may apply to other study areas with higher Hg loading and so reinforce the importance of studying interactions between behavior, season, and habitat.

## 5 CONCLUSIONS

We used stable isotopes of carbon and nitrogen to determine how the riparian forest songbird communities relied on aquatic energy subsidies. These species (Common Yellowthroat, Spotted Towhee, Swainson's Thrush, Song Sparrow, and Yellow Warbler) or closely related conspecifics are widespread throughout North American riparian areas and represent an understudied community in aquatic contaminant studies. The species we studied varied in their reliance on aquatic prey and subsequent Hg exposure at both a species and individual level. We were not only able to correlate Hg concentrations in songbirds to their reliance on aquatic‐based prey, but also showed that birds forage on more aquatic‐sourced prey early in the season than later. These findings suggest that pulsed emergence of aquatic invertebrates may be an important vector of Hg to avian insectivores. These findings are particularly relevant in the face of climate change, which can alter the timing and magnitude of emergent aquatic subsidies (Larsen et al., 2016).

## CONFLICT OF INTEREST

The authors have no conflicts of interest.

## AUTHORS CONTRIBUTION

**Allyson Kathleen Jackson:** Conceptualization (lead); Formal analysis (lead); Funding acquisition (supporting); Investigation (lead); Methodology (equal); Visualization (lead); Writing‐original draft (lead); Writing‐review & editing (equal). **Collin A. Eagles‐Smith:** Conceptualization (supporting); Formal analysis (supporting); Funding acquisition (lead); Methodology (equal); Resources (lead); Supervision (equal); Writing‐review & editing (equal). **W Douglas Robinson:** Conceptualization (supporting); Methodology (supporting); Supervision (equal); Writing‐review & editing (equal).

## Data Availability

Jackson, A.K., Eagles‐Smith, C.A., and Robinson, W.D., 2021, Mercury Concentrations and Stable Isotopes in Riparian Songbirds and Invertebrates from the Willamette River, Oregon, 2013: U.S. Geological Survey data release, https://doi.org/10.5066/P9FD0GOV
